# Impact of the Dicarboxylic Acid Chain Length on Intermolecular Interactions with Lidocaine

**DOI:** 10.1021/acs.molpharmaceut.2c00381

**Published:** 2022-07-19

**Authors:** Julija Zotova, Brendan Twamley, Lidia Tajber

**Affiliations:** † School of Pharmacy and Pharmaceutical Sciences, Trinity College Dublin, College Green, Dublin 2, Ireland; ‡ School of Chemistry, Trinity College Dublin, College Green, Dublin 2, Ireland

**Keywords:** dicarboxylic acid, lidocaine, eutectic, phase diagram, ionization

## Abstract

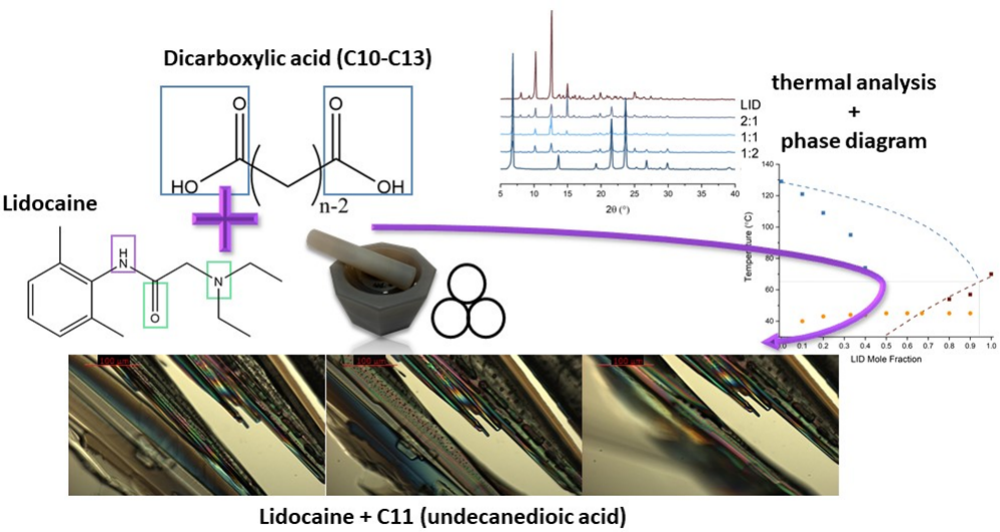

Acid–base multicomponent systems have become a popular choice as a strategy to fine-tune the physicochemical properties of active pharmaceutical ingredients. Current prediction tools based on the principles of anticrystal engineering cannot always accurately predict the nature of intermolecular interactions within a multicomponent system. Even small changes in the physicochemical parameters of parent components can result in unexpected outcomes, and many salt, cocrystal, and ionic liquid forms are still being discovered empirically. In this work, we aimed to establish structural consistency in a series of mixtures comprising lidocaine (LID) with decanedioic, undecanedioic, dodecanedioic, and tridecanedioic acids and to explore how length and flexibility of the acid carbon backbone affect the molecular recognition, crystallization, and thermal behavior of the expected binary systems. We found that neat grinding of LID with dicarboxylic acids results in the formation of eutectic phases. The observed eutectic melting points deviated from the ideal eutectic temperatures predicted by the Schroeder van Laar model because of hydrogen bonding between the reacting components within the mixtures. Furthermore, thermal and infrared analysis provided evidence for the possible formation of new phases stemming from partial ionization of the counterions. Besides, the structure of a previously undetermined form I of the tridecanedioic acid was solved by single crystal X-ray diffraction.

## 1 Introduction

Active pharmaceutical ingredients (APIs) can exist in a variety of morphological forms ranging from solids to liquids, from crystals to amorphous materials, and from single molecular structures to complicated multicomponent systems.^1,2^ Moreover, they can be found in a wide spectrum of ionization states comprising fully ionized salts, partially ionized and “confused” eutectic solvents, and neutral cocrystals, among a vast array of others.^3,4^ Altering and fine-tuning of the physical form of APIs can greatly affect drug formulation strategies and drug performance.^5^

Principles of crystal engineering have permitted a more systematic approach to be taken to engineer the desired properties of pharmaceutical materials.^6−8^ These principles have been employed in a new concept, called anticrystal engineering, to obtain alternative forms of APIs with inhibited crystallization.^9,10^ Anticrystal engineering had initially been defined as a method of identifying and intentionally avoiding the pairing of cations and anions with the goal of inhibiting salt crystallization.^9^ Later, it had been shown that the presence of hydrogen bonds is fundamental in the liquefaction of multicomponent phases, and subsequently, the scope of anticrystal engineering has expanded to include neutral nonionic species.^11,10^ Other factors, such as low symmetry and charge delocalization, contribute to the inefficient packing of counterions or coformers, thereby contributing to a decreased likelihood of crystallization.^12^

However, current prediction tools cannot always accurately predict the nature of a multicomponent system because slight changes in the physicochemical parameters of parent components lead to significantly different outcomes, and many salt, cocrystal, and ionic liquid forms are still being discovered empirically.^13^ One of the popular discovery approaches employed is mechanochemical screening. It involves a variety of techniques, including neat grinding, solvent-assisted grinding, and milling.^14−18^ In addition to its ease of experimental design and high screening efficiency, mechanochemical synthesis is also generally considered as a green alternative to conventional synthetic methods because of its minimization of organic solvent use and reduced need for product purification steps.^19^

We have previously employed an anticrystal engineering approach to investigate the effect of a range of crystal and physicochemical parameters in the formation of multicomponent systems comprising lidocaine (LID) and a series of short- and medium-chain aliphatic dicarboxylic acids (DA).^20^ LID, 2-(diethylamino)-N-(2,6-dimethylphenyl)acetamide, is an aminoamide drug used as an active pharmaceutical ingredient in local anesthetic formulations. It is often selected as a model compound for investigation of the physicochemical properties of a multicomponent phase formation.^21,11,22,23^ In previous work on the LID:DA systems, we have observed alternating trends in morphology, melting points, glass transition temperatures, and crystallographic properties of the new phases across the dicarboxylic acid series.^20^ In addition, we have investigated the influence of a changing counterion ratio on the nature of intermolecular interactions and the identity of new phase formation.^24^ Upon addition of excess LID base component to azelaic acid, a neutral cocrystal multicomponent phase was obtained. Alternatively, the presence of excess acid component has resulted in the formation of a range of proton-transferred ionic liquids.^24^

In this work, we are diving deeper into the aliphatic dicarboxylic acid homologous series with the aim to establish structural consistency in the extended series of LID:DA systems and to explore how the length and the flexibility of the long carbon backbone affect the molecular recognition, crystallization, and thermal behavior of the expected binary systems. Decanedioic (sebacic), undecanedioic, dodecanedioic, and tridecanedioic acids used in the current study have been symbolized as C10, C11, C12, and C13, where a number denotes the number of carbon atoms in the acid backbone. Such a designation has been adopted to follow a series of works investigating the even–odd alternation of physicochemical properties of saturated aliphatic dicarboxylic acids.^25−27^ While shorter DAs are a popular choice for use as constituents in multicomponent systems,^28,29^ longer-chain DAs are much less explored. C10 has been employed for the formation of multicomponent phases in combination with propranolol,^30^ apatinib,^31^ and isonicotinamide.^32^ C12 has been investigated as a possible coformer in combination with praziquantel^33^ and pyridinecarboxamide-based potential cancer drugs.^34^ To the best of our knowledge, API:C11 or API:C13 systems have not been reported to date. Similar to the multicomponent systems outlined above, the possible application of LID:DA based on C10–C13 could be to improve solubility/dissolution rates and/or improve skin penetration.

## 2 Materials and Methods

### 2.1 Materials

LID (as a base), sebacic acid (C10), dodecanedioic acid (C12), and 1,11-undecanedicarboxylic acid (C13) were purchased from Aldrich (Ireland). 1,9-Nonanedicarboxylic acid (C11) was purchased from TCI (Belgium). HPLC grade ethanol was obtained from Fisher Chemical (Ireland). All chemicals were used as supplied.

### 2.2 Methods

#### 2.2.1 Sample Preparation

The binary mixtures were prepared by accurately weighing LID and each of the acids using a Mettler Toledo MT5 microbalance (Mettler Toledo, Switzerland) at a range of molar fractions. The powders were then coground using an agate pestle and mortar until homogeneous mixtures were obtained. The samples were transferred into microcentrifuge tubes and were kept at 4 °C for 24 h before being analyzed.

#### 2.2.2 Differential Scanning Calorimetry (DSC)

DSC scans were carried out using a PerkinElmer Pyris Diamond DSC unit (USA) equipped with a ULSP B.V. 130 cooling system (Netherlands). Approximately 3–5 mg of each sample were accurately weighed using a Mettler Toledo MT5 microbalance (Mettler Toledo, Switzerland) into aluminum pans and sealed. The samples were subjected to a first heating cycle at a 10 K min ^–1^ rate, followed by a fast-cooling step to −60 °C at a 300 K min^–1^ rate, and finally a second heating cycle at a 10 K min^–1^ rate. N_2_ gas at a flow rate of 40 mL min^–1^ was employed as a purge gas. The experimental control and data analysis were performed using PerkinElmer Pyris software. The samples were measured at least in duplicates.

#### 2.2.3 Polarized Optical Microscopy (POM)

Prior to analysis, LID and C11 were dissolved in ethanol left at room temperature until complete solvent evaporation. A small crystal of each LID and C11 were placed on a glass slide in direct contact with one another. The solid fusion experiment was monitored using an Olympus BX53 polarizing optical microscope (Mason Technologies, Ireland) equipped with a U-POT cross polarizer and a U-AND analyzer. Images were taken with an integrated Q IMAGING Fast 1394 camera (Olympus, Japan). The experiment was performed at room temperature, which was recorded at 22.7 ± 0.5 °C.

#### 2.2.4 Powder X-ray Diffraction (PXRD)

Room-temperature PXRD was performed using a Rigaku Miniflex II X-ray diffractometer (Japan). Cu Kα (1.54 Å) was used as a radiation source. The scan rate of 0.05°/s at the range of 2–40 2θ degrees was employed. The tube voltage and tube current used were 30 kV and 15 mA, respectively. Freshly ground LID:DA in a range of molar compositions were analyzed. In addition, equimolar LID:DA compositions were heated in an oven at 60 °C until molten, followed by slow cooling and drying under vacuum at room temperature for 60 h. The melt–cool samples were then analyzed by PXRD.

#### 2.2.5 Attenuated Total Reflectance Fourier-Transform Infrared Spectroscopy (ATR-FTIR)

ATR-FTIR spectra were obtained with a Spectrum One spectrophotometer (PerkinElmer, USA) equipped with a diamond ATR accessory and Spectrum v.5.0.1 software. The samples were dried in an oven at 60 °C for 30 min prior to analysis. A spectral range of 650–4000 cm^–1^ and accumulation of 8 scans were used. Deconvolution of the infrared bands was conducted using the Gauss function fitting module in OriginLab ver. 7.5.

#### 2.2.6 Single Crystal X-ray Diffraction (SXRD)

A powdered sample of LID:C13 at 1:1 molar ratio was dissolved in a minimum amount of ethanol and was allowed to stand at room temperature until complete solvent evaporation. Colorless crystals of the C13 acid of satisfactory quality for SXRD analysis were obtained.

Data for the C13 were collected on a Bruker APEX DUO using Cu Kα radiation (λ = 1.54178 Å). The sample was mounted on a MiTeGen cryoloop, and data was collected at 100(2) K using an Oxford Cobra cryosystem. Bruker APEX^35^ software was used to collect and reduce the data. Absorption corrections were applied using SADABS.^36^ Structures were solved with the SHELXT structure solution program^37^ using Intrinsic Phasing. The data were refined using the Least Squares method on F2 with SHELXL.^38^ All non-hydrogen atoms were refined anisotropically. Hydrogen atoms were assigned to calculated positions using a riding model with appropriately fixed isotropic thermal parameters. Molecular graphics were generated using OLEX2.^39^ The crystal data, details of the data collection, and their refinement are given in Table S1.

The acid group hydrogen H1 was located on the difference map and refined.

The crystallographic data for the structure in this paper have been deposited with the Cambridge Crystallographic Data Centre as supplementary publication no. 2168827. Copies of the data can be obtained, free of charge, on application to CCDC, 12 Union Road, Cambridge CB2 1EZ, UK (fax: + 44-(0)1223–336033) or e-mail:deposit@ccdc.cam.ac.uk.

#### 2.2.7 Crystallographic Analysis

Mercury (version 2020.2.0)^40^ was used for structure visualization, crystal packing analysis, molecule overlay calculations, and unified pair-potential (UNI) force-field calculations.^41,42^ The reference structures used for calculations were obtained from the Cambridge Structural Database (CSD): LIDCAN11,^43^ SEBAAC06,^27^ UNDEAC11,^44^ UNDEAC12,^44^ DECDAC02,^45^ and BRASAC11.^46^ Void spaces within crystal lattices were calculated using a contact surface with probe radius of 0.2 Å and an approximate grid spacing of 0.2 Å.

#### 2.2.8 Density Functional Theory (DFT) Calculations

Gaussian03 program was employed to calculate the total energy of the fully optimized structures of C10, C11, C12, and C13, extracted from the single crystal structures SEBAAC06,^27^ UNDEAC11,^44^ UNDEAC12,^44^ DECDAC02,^45^ BRASAC11,^46^ and the new form of C13 obtained in this work.^47^ The B3LYP/6-31++G(d,p) level of density functional theory (DFT) was used. Wave function analysis was conducted using Multiwfn 3.6, as described previously.^10,48,49^

## 3 Results and Discussion

### 3.1 Anticrystal Engineering Considerations

**Scheme 1:**
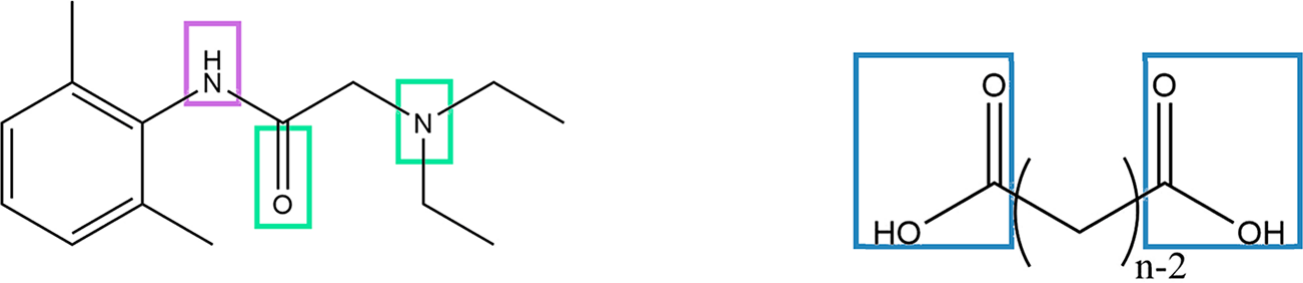
Molecular Structures of LID (Left) and a General Molecular Structure of a DA (Right), where *n* = Number of Carbon Atoms in the Backbone, Purple = H-Bond Donor and Acceptor, Green = H-Bond Acceptor, and Blue = Carboxylic Acid Groups

LID is a weak base with a p*K*_a_ of approximately 8.0.^50^ It possesses one H-donor and three H-acceptor groups, as shown in Scheme 1. Upon sufficient difference in p*K*_a_ (Δp*K*_a_) values, which is defined as Δp*K*_a_ = p*K*_a__,base_ – p*K*_a,__acid_, LID may form molecular salts via ionization at the tertiary amine site. The sufficient Δp*K*_a_ is commonly accepted to be >3 units.^51^ The p*K*_a_ value for C10 was experimentally determined and the p*K*_a_ values for C11, C12, and C13 were theoretically calculated to be 4.7,^52−55^ thus generating sufficient Δp*K*_a_ to facilitate proton transfer. However, due to the flexibility and bulkiness of DAs, the access to the H-bonding active sites may be sterically hindered. As a result, the degree of proton transfer may not be derived theoretically in a reliable fashion, and experimental characterization is crucial for the characterization of multicomponent phases. The principles of molecular complementarity applicable to crystal engineering complicate the possibilities of molecular interactions further. It is well known that acid carboxyl moieties tend to form supramolecular heterosynthons with amide moieties of basic compounds.^56^ Therefore, LID possessing an amide group has a potential to form additional hydrogen bonds via amide-carboxyl synthon formation.

In an anticrystal engineering approach, the aim of a counterion or a coformer is to lower the melting point (*T*_m_) of an API. It has been shown that, in general, the *T*_m_ of a salt or a cocrystal is influenced by the melting point of the parent materials.^13,57^ The *T*_m_ of the DAs investigated alternates with an even–odd number of carbons in the carbon backbone. The thermal data in Table 1 was obtained for the parent DAs and is in good agreement with previously published data.^58^ Therefore, it is anticipated that the melting points of any multicomponent systems formed will also alternate in a similar fashion.

**Table 1:** Physicochemical and Crystallographic Properties of the Investigated Molecules^a^

molecule	molecular weight(g/mol)	melting point (° C)	Δ*H* (kJ/mol)	polymorphic form	KPIs (%)	packing energy (kJ/mol)	ref.
LID	234.34	69–70	16.0	*P* 2_1_/*c*	70.2		(43)
C10	202.25	133–136	45.4	*P* 2_1_/*c*	70.7	–156.5	(27)
C11	216.28	109–112	43.7	*C* 2/*c* (form I)	76.4	–174.4	(44)
				*P* 2_1_/*c* (form II)	75.6	–172.8	(44)
C12	230.3	129–131	51.6	*P* 2_1_/*c*	71.3	–171.7	(45)
				*C* 2/*c* (form I)	76.1	–189.6	this work
C13	244.33	112–115	52.9	*P* 2_1_/*n* (form II)	74.5	–187.0	(46)

a Melting point and enthalpy of fusion (Δ*H*) data was determined by DSC analysis. Kitaigorodskii Packing Indices (KPIs) were calculated from the void space measurements. It was not possible to calculate the packing energy of LID because of the disorder present in the crystal structure

Higher torsion angles, twisting of the carbon chain, and overall greater distortion from the equilibrium conformation of the odd dicarboxylic acids lead to energetically unfavorable molecular conformations.^25^Table 1 presents a summary of crystallographic properties of LID and DAs, their determined polymorphic forms and corresponding packing energies. It is hypothesized that the energy difference between the solid state and an ideal isolated state released upon grinding or melting could influence the tendency of the acids to form multicomponent systems with bases. It means that, upon mechanochemical synthesis, odd-chain DAs would display preference for forming multicomponent phases with LID rather than retaining or reforming highly strained parent acid structures. We have previously proven this hypothesis, where LID:DA systems with glutaric, pimelic, and azelaic acids displayed poor crystallization tendency and readily formed ionic liquids upon physical contact. In contrast, LID with adipic acid required heat treatment for the formation of lidocaine hemiadipate salt, and LID with suberic acid formed a mixture of acid–base eutectic and lidocaine hemisuberate salt upon grinding.^20,24^

Kitaigorodskii’s Packing Indices (KPIs) are another useful measure that can aid in the prediction of counterion interaction tendency.^59^ KPIs are linked with the theory that parent materials with less efficient packing have larger cavities, thereby providing an enhanced availability of active sites required for solid state reactions.^59^ According to these postulates, less efficiently packed DAs (i.e., characterized by lower KPIs) are predicted to exhibit a greater tendency to undergo solid state reactivity upon grinding and should form multicomponent systems more readily.

Molecular modeling provided further information in relation to the probable behavior of the DAs. Mapping of the calculated electrostatic potential (ESP) on the molecular vdW surface of the DAs (C10–C13) showed that the minimum ESP values were comparable and ranged from −38.00 to −36.50 kcal/mol (Table 2). The location of this minimum was on the carbonyl moiety of the −COOH group, which indicates its electrophilic nature. The maxima were slightly more dissimilar and varied between 49.09 and 53.92 kcal/mol with said maxima located on the hydrogen atom of the −OH moiety of the carboxylic group and representing the nucleophilic part of the molecule. With these values, the DAs are expected to interact with LID via hydrogen bonds driven by electrostatic attraction between the molecules.^60,10^ However, considering the values of σ_tot_^2^, C10 and C12 should interact with LID more strongly, because the higher the σ_tot_^2^ value, the stronger the tendency to interact electrostatically with another molecule (Table 2).^61^

**Table 2:** Values of General Interaction Properties Function (GIPF) Descriptors, As Defined by the Molecular vdW Isosurface at ρ = 0.001 e/Bohr^3^ and *V*_min_ – Global ESP Minimum, *V*_max_ – Global ESP Maximum, and σ_tot_^2^ - Total ESP Variance

DA	*V*_min_ (kcal/mol)	*V*_max_ (kcal/mol)	*σ*_tot_^2^ (kcal/mol)^2^
C10	–38.00	53.92	235.2
C11 (form I)	–35.91	49.17	189.5
C11 (form II)	–37.55	49.57	192.7
C12	–37.83	53.01	232.0
C13 (form I)	–36.50	50.51	196.8
C13 (form II)	–37.41	49.09	188.0

In summary, on the basis of the KPIs, C11 and C13 should display a greater tendency to undergo solid-state reactivity considering the grinding process, while C10 and C12 should interact with LID more strongly and, thus, form multicomponent systems more readily.

### 3.2 Mechanochemical Synthesis and Crystallization Studies

**Figure 1:**
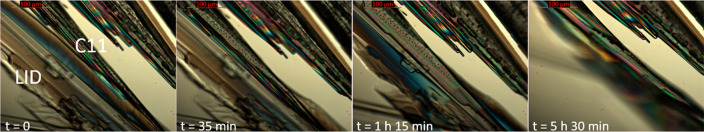
A solid fusion experiment performed with recrystallized LID (bottom left: plate-like crystal) and recrystallized C11 (top right and center: needle-like crystals). After 5 h 30 min, most of the LID has dissolved within the eutectic, while most of C11 that has not been in direct contact with LID has remained unchanged.

Upon manual neat grinding, homogeneous binary mixtures of LID:DAs in the form of white solid powders were obtained. Mixtures of LID:C11 in the range of χ_LID_ = 0.67–0.33 molar fractions were obtained as a white semiliquid paste. A solid fusion experiment performed using POM revealed that single crystals of LID and C11 undergo solid interdiffusion at room temperature and form a liquid eutectic at temperatures below 23 °C, as evident in Figure 1. The observation resembles the solid-state reactivity of LID with glutaric, pimelic, and azelaic acids.^20,24^

**Figure 2:**
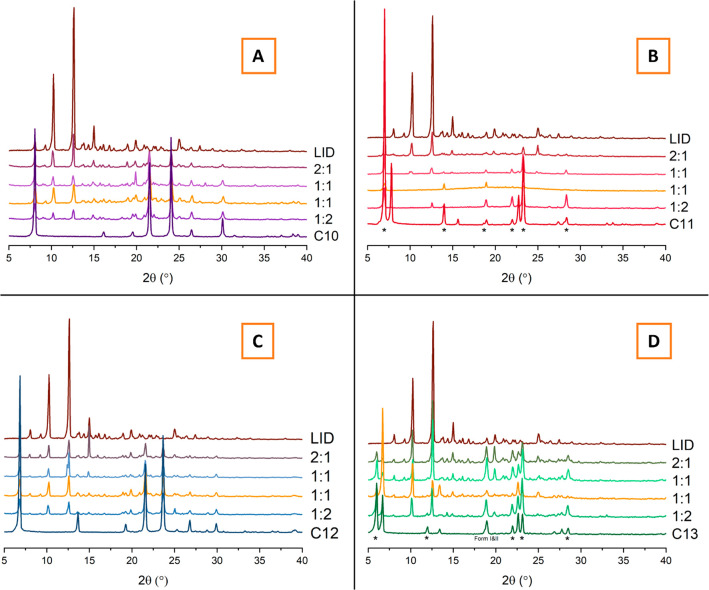
Stacks of PXRD diffractograms at different molar compositions of (A) LID:C10; (B) LID:C11, where stars under the diffractogram of the pure C11 sample denote peaks corresponding to the *C* 2/*c* polymorph (form I) and unmarked peaks correspond to the *P* 2_1_/*c* polymorph (form II); (C) LID:C12; and (D) LID:C13, where stars under the diffractogram of the pure C13 sample denote peaks corresponding to the *C* 2/*c* polymorph (form I) and unmarked peaks correspond to the *P* 2_1_/*n* polymorph (form II). Diffractograms marked in orange represent equimolar samples that were heated until completely molten and then dried under vacuum at room temperature for 60 h.

PXRD analysis of the parent materials and freshly ground binary mixtures reveals combinations of the parent diffractograms without new Bragg peaks in any of the four systems (Figure 2A–C). Thus, no new crystalline phases were created upon neat grinding. The binary mixtures at a 1:1 molar ratio were then heated at 60 °C until molten, followed by slow cooling and drying under vacuum at room temperature in an attempt to promote the formation of intermolecular interactions. The melt–cool crystallization experiments yielded diffractograms with a combination of peaks stemming from the parent materials. The diffractograms for the melt–cool samples are highlighted in orange color in Figure 2. However, it is important to note that partial liquefaction of LID:C11 samples in the range of χ_LID_ = 0.67–0.33 molar fractions provides a likely indication for a new phase formation characterized by a melting point that is lower than room temperature. Solvent-evaporation crystallization from water, methanol, ethanol, and acetonitrile also proved to be unsuccessful, and only parent dicarboxylic acids have crystallized out. This contrasts with the observations made for shorter-chain LID:DA systems with oxalic, malonic, succinic, glutaric, adipic, pimelic, suberic, and azelaic acids, which were obtained by both mechanochemistry and solution-based methods.^62,20,24^

**Figure 3:**
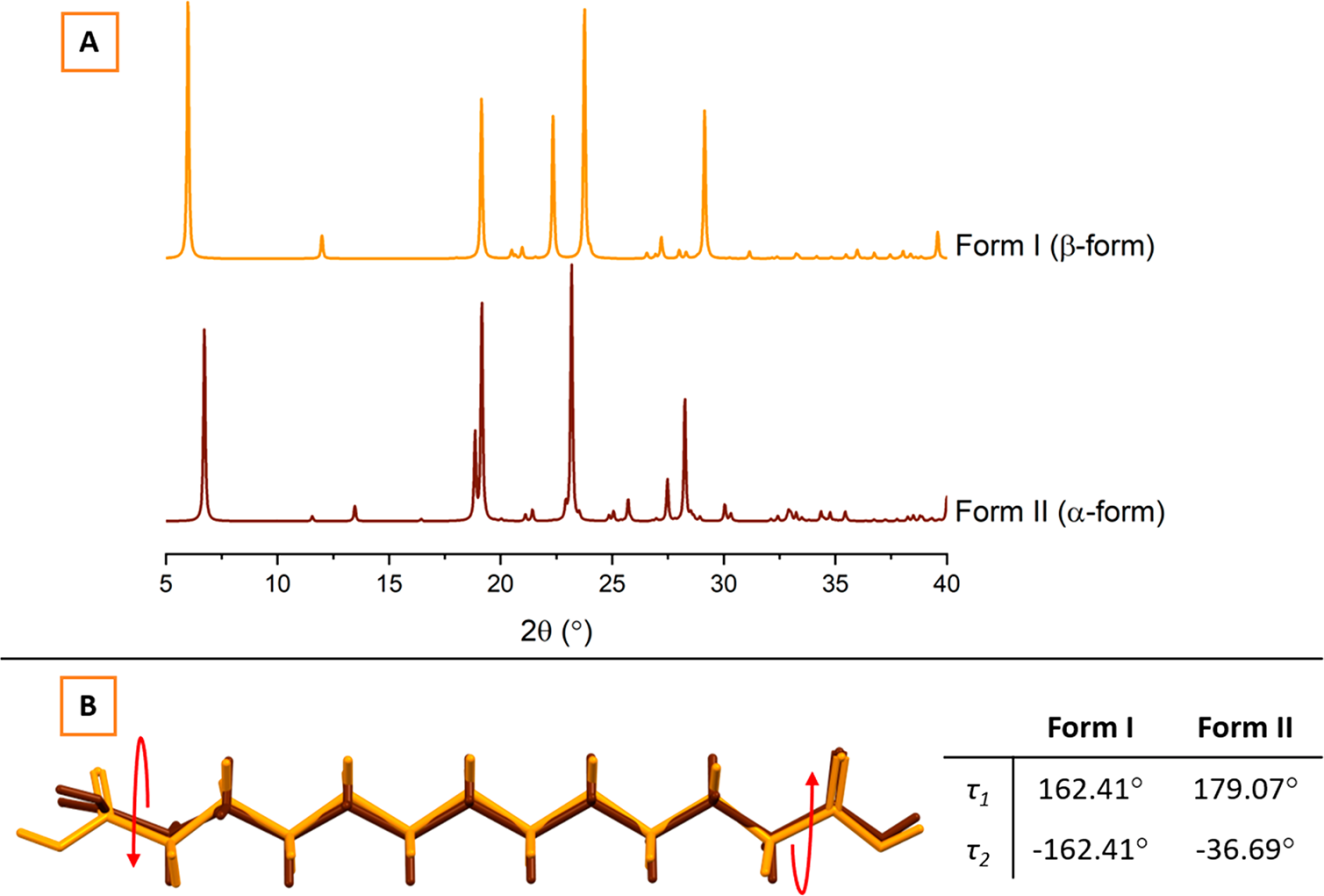
(A) Calculated PXRD patterns of forms I and II of C13 dicarboxylic acid. Crystal structure of form II was obtained from CSD.^46^ (B) Molecular overlay of form I and form II of the C13 dicarboxylic acid displaying the differences in torsion angles.

During crystallization efforts, a previously undetermined low-temperature form I (β-form) of C13 acid was isolated and analyzed by SXRD. Full crystallographic characterization may be found in Table S1. The high-temperature form II (α-form), however, has been previously determined and characterized by multiple research groups.^63,46^ The PXRD patterns of the two forms are presented in Figure 3A.

It is interesting to note that medium and long odd-chain dicarboxylic acids with a carbon chain length in the range of C7–C15 only crystallize as a form II from solvents with H-bond-donating ability.^46^ However, in the current study, the presence of LID molecules in ethanolic solution has opposed the solvent-dependent polymorph selectivity pattern within the acid homologous series, and form I of C13 has crystallized out.

**Figure 4:**
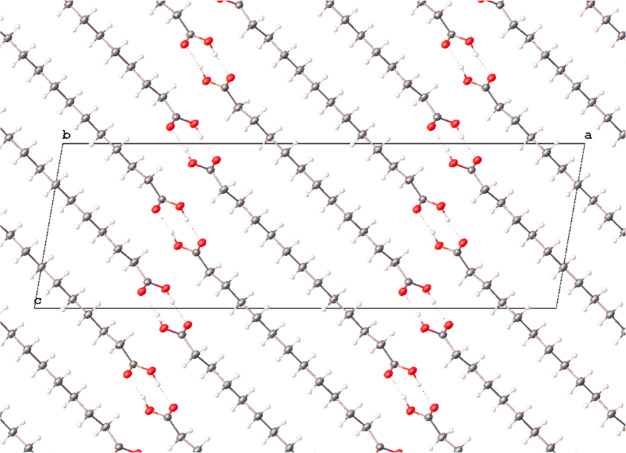
Packing diagram of form I of C13 viewed normal to the *b* axis showing the acid hydrogen bonding motif. Displacement at 50% probability.

Analogous to other odd-chain dicarboxylic acids within the homologous series, form I of C13 crystallizes as a monoclinic crystal system and is assigned to the *C* 2/*c* space group. The molecules within the crystal are arranged as carboxylic acid dimers, formed via OH···O hydrogen bonding with *d*(D···A) = 2.6619(16) Å, which are further assembled into stacked molecular chains, as shown in Figure 4. The unfavorable O···O repulsion between the carboxyl groups of the neighboring chains are avoided because the C13 molecules are found in a twisted conformation with torsion angles for O_1_–C_1_–C_2_–C_3_ and O_1_^i^–C_1_^i^–C_2_^i^–C_3_^i^ being τ_1_ = τ_2_ = ± 162.41(10)°. In contrast, the torsion angles in the form II are different at both ends of the molecule, as shown in Figure 3B. This observation is consistent with the general trend followed by the series of odd-chain dicarboxylic acids.

### 3.3 Thermal Analysis of the Binary Mixtures

The binary mixtures at a range of molar compositions were analyzed by DSC, and the data obtained from the first heating cycle was used to construct thermodynamic phase diagrams. Theoretical solubility curves were constructed using the Schroeder van Laar equation (eq 1), where *T*_fus_ (K) is the temperature of fusion and Δ*H*_fus_ (J mol^–1^) is the enthalpy of fusion of the pure starting component, χ is the mole fraction of the pure starting component at a specified temperature *T* (K), and *R* denotes the universal gas constant. The Schroeder van Laar model describes the solid solubility behavior of the starting components that do not interact and form an ideal eutectic mixture.^64^
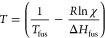
1

The thermal data for the parent materials presented in Table 1 was obtained from the first heating cycle. Namely, LID, C10, and C12 displayed a single endothermic transition at 69, 133, and 129 °C, respectively. These transitions were ascribed to the melting events of the pure materials that are only found as a single polymorphic form. Endothermic transitions for the C11 and C13 dicarboxylic acids at 109 and 112 °C, respectively, appeared as merged peaks because of the odd dicarboxylic acids possessing two distinct polymorphic configurations each.^46,58^ However, because of the closeness of the melting points of the different polymorphs it is not possible to separate the overlapping peaks.

**Figure 5:**
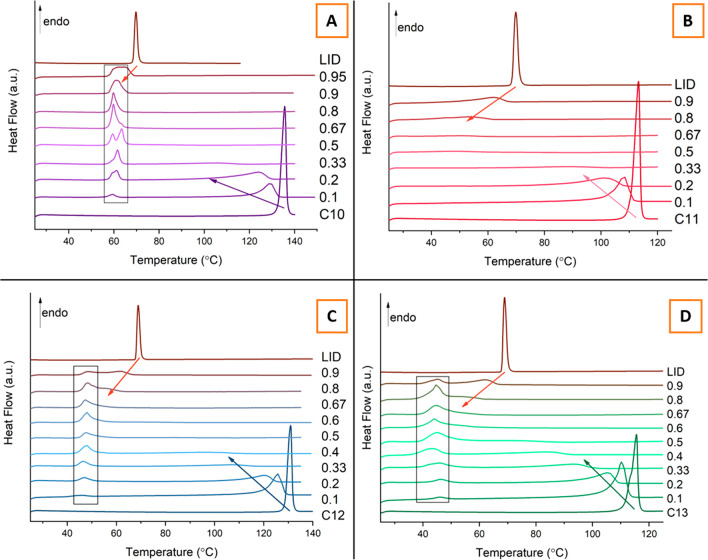
Stacks of 1st heating cycle DSC thermograms at different molar compositions of (A) LID:C10, (B) LID:C11, (C) LID:C12, and (D) LID:C13. Red arrows indicate the LID melting point depression with increasing DA molar fraction. Purple, pink, blue and green arrows indicate the melting point depression of C10, C11, C12, and C13, respectively.

**Figure 6:**
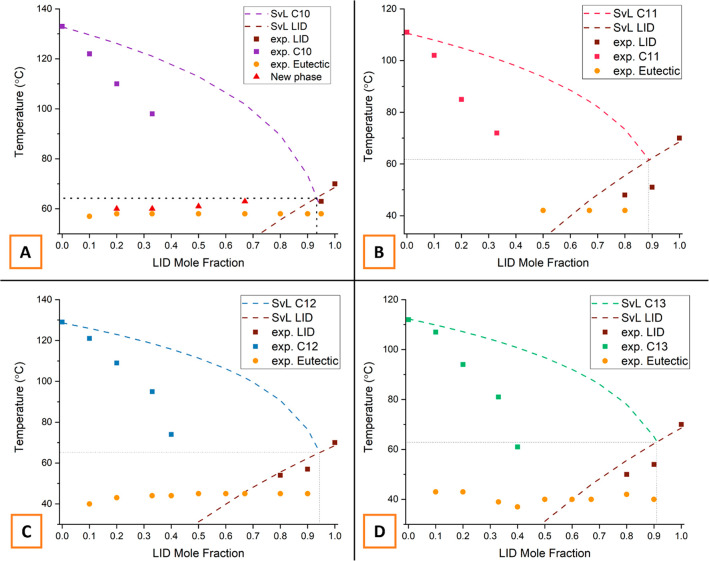
Phase diagrams of the four systems studied: (A) LID:C10, (B) LID:C11, (C) LID:C12, and (D) LID:C13. Dashed lines were calculated using the Schroeder van Laar (SvL) equation. Dotted lines represent the theoretical temperature and composition of the eutectic as determined by the Schroeder van Laar equation.

In all of the four systems studied and presented in Figures 5 and 6, the expected melting point depression of the parent materials is observed. However, the extent of the melting point depression deviates from the ideal solubility curve predicted by the Schroeder van Laar model. This deviation from ideality implies the formation of intermolecular interactions between the parent components that contribute to the further lowering of the melting point. Eventually, at certain molar compositions, no peaks are observed for residual LID and dicarboxylic acid endothermic transitions.

The analysis of LID:C10 binary mixtures presented in Figures 5A and 6A indicate the presence of two distinct endothermic peaks at 58 and 61 °C, most visible at the 1:1 molar composition. A slight shifting of the endotherm at 61 °C in the samples with a higher molar fraction of C10 acid suggests that it may, indeed, be a new distinct phase, such as a salt or a cocrystal. Such a double peak phenomenon was observed in LID systems with other even-chain DAs, adipic and suberic, where the peaks were attributed to a salt and a eutectic melting event, respectively.^20^ In both systems, LID:adipic acid and LID:suberic acid, the crystal structures of the fully ionized salts were solved. However, crystallization efforts of the LID:C10 system did not yield definite evidence for the formation of such a phase. Binary mixtures of the LID:C11 system also exhibit a eutectic formation in the samples with a higher molar fraction of LID, as seen in the phase diagram in Figure 6B. The peaks for the fusion events of the eutectic appear at 42 °C but are not visible in the stack of the DSC traces in Figure 5B because of the peak broadness and low Δ*H*_fus_ values. A stack of DSC thermograms with the magnified 40–60 °C region is presented in Figure S1. This observation is not unusual for the semiliquid paste sample morphology and has been observed in other LID:DA systems with odd-chain dicarboxylic acids.^20^ It can be explained by the dispersion of the solid particles within a liquid matrix that gradually dissolves upon melting as a result of slower heat transfer processes. LID:C12 and LID:13 exhibit similar thermal behavior, as shown in Figures 5C–D and 6C–D. A single eutectic is formed in both systems at 45 and 40 °C, respectively.

The melting points of the eutectics in all of the four systems studied exhibit deviation from the theoretical eutectic temperature predicted by the Schroeder van Laar model. One of the suggested definitions of a “deep eutectic solvent” states that DES is a mixture of pure compounds for which the eutectic point temperature is below that of an ideal liquid mixture.^64^ The melting point depression between the ideal and the observed eutectic point is used to measure how “deep” the eutectic is. The calculated deviations for the systems studied summarized in Table 3 show that only LID:C10 could be reasonably predicted as the differences in the eutectic melting points for other systems are ≥20 °C. Analogous to the melting point depression of the parent components, the formation of intermolecular interactions, such as hydrogen bonds, within the eutectics contribute to the observed “deepening” of the melting points.^65^ Therefore, FTIR spectroscopy, as described below, was employed to investigate the nature of these intermolecular interactions. It is interesting to note that even though the observed eutectic points follow the expected even–odd alternating pattern, the calculated deviations Δ*T*_e_ do not. The Schroeder van Laar model can also predict ideal eutectic composition, χ_e_. The theoretical χ_e_ calculated for the LID:DA systems also exhibit even–odd alternating pattern, although the values are very similar, see Table 3.

**Table 3:** Calculated Differences Δ*T*_e_ between the Predicted Ideal Eutectic Melting Temperature *T*_e, ideal_ and an Observed Experimental Eutectic Melting Temperature *T*_e, observed_

system	χ_e, ideal_	*T*_e, ideal_ (° C)	*T*_e, observed_ (° C)	Δ*T*_e_ (° C)
LID:C10	0.93:0.07	64	58	6
LID:C11	0.89:0.11	62	42	20
LID:C12	0.94:0.06	65	45	20
LID:C13	0.91:0.09	63	40	23

**Figure 7:**
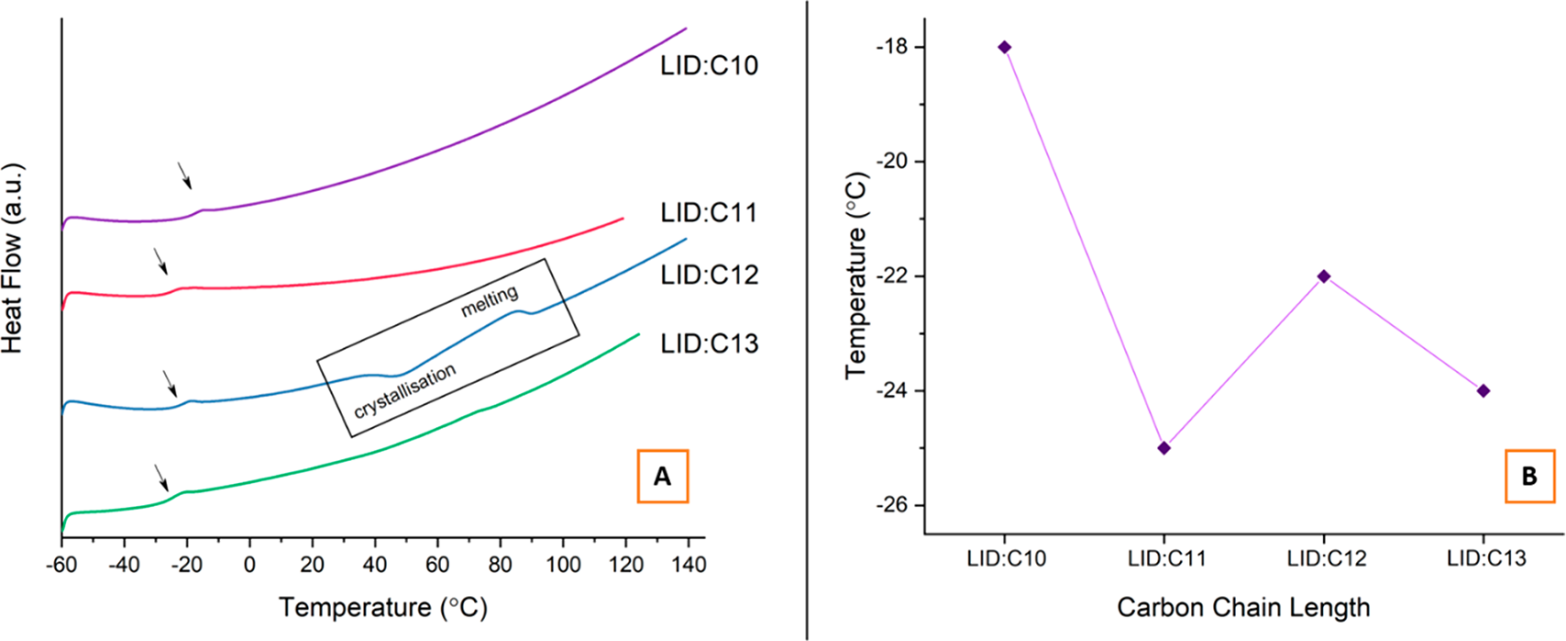
Alternating trend in *T*_g_ values of the equimolar LID:DA mixtures.

After the first heating, the 1:1 LID:DA samples were fast cooled at a nominal rate of 300 °C/min and subjected to a second heating cycle. Figure 7A presents a stack of DSC thermograms for the second heating cycle, which reveal the good glass-forming ability of LID:DA equimolar samples. It is manifested by their ability to be supercooled, i.e., no melting or crystallization events are observed upon cooling and/or before heating to the glass transition temperature (*T*_g_). In contrast, pure starting components do not exhibit *T*_g_ transitions as they crystallize during the cooling step. The observed *T*_g_ values of the equimolar binary mixtures follow the usual even–odd alternating pattern, as shown in Figure 7B. It has been suggested that the nature of intermolecular interactions in the supercooled state impacts the position of the *T*_g_.^66^ Therefore, the 1:1 LID:C10 system characterized by the highest *T*_g_ value of −18 °C is expected to form the strongest electrostatic interactions in the supercooled state, and LID:C11 with the *T*_g_ value of −25 °C forms the weakest. A similar trend has been observed for shorter dicarboxylic acid (C5–C8):LID equimolar supercooled mixtures.^20^ However, the nature of the proton-transfer mechanism and the presence of other intermolecular interactions (e.g., van der Waals forces) can also shift the position of the *T*_g_.^67^

In addition, the 1:1 LID:C12 sample also exhibits crystallization and melting peaks upon heating past the glass transition temperature. PXRD analysis of the crystallized material revealed that it was composed of both LID and C12 parent materials (Figure 2c).

### 3.4 Investigation of Intermolecular Interactions

**Figure 8:**
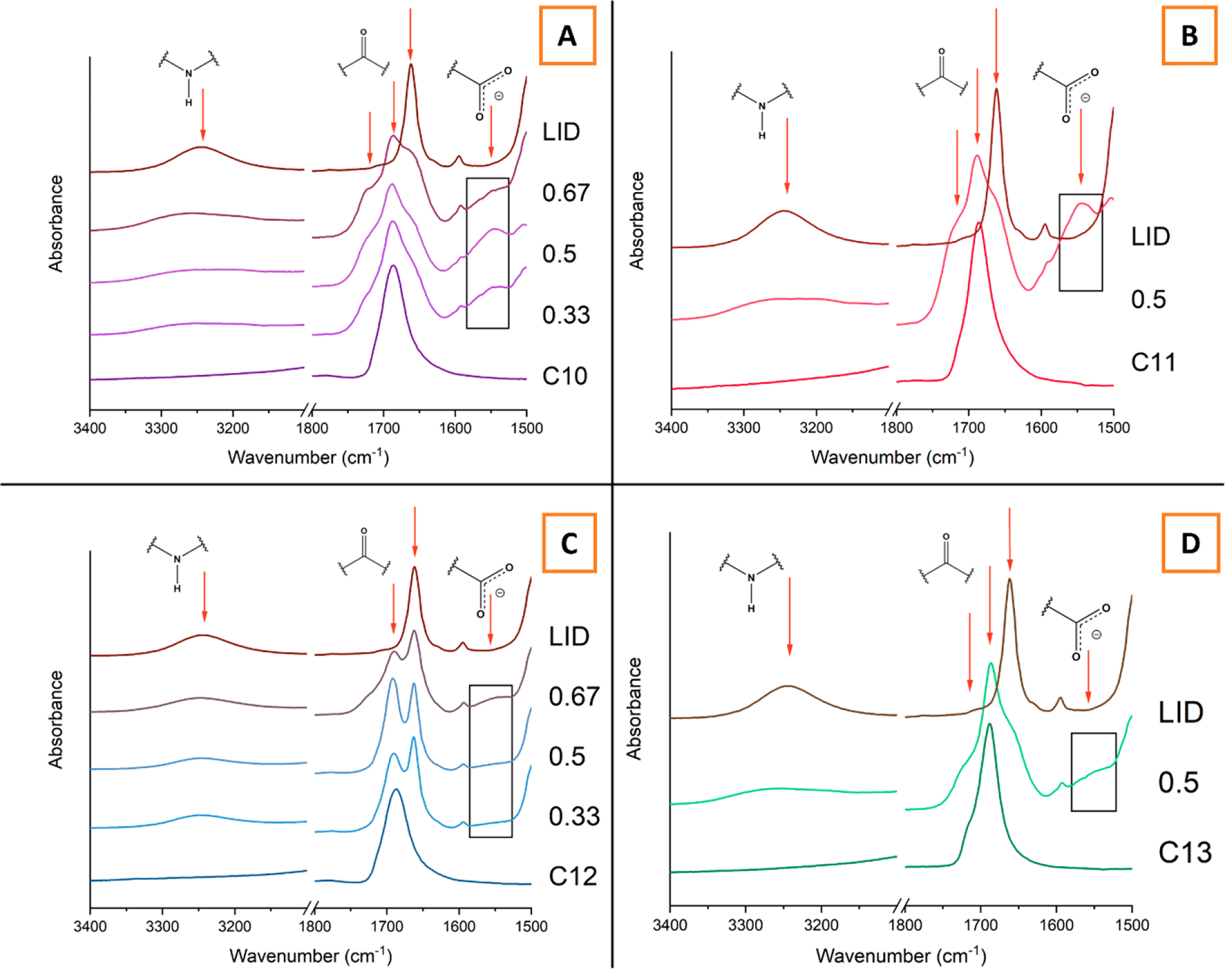
Stacks of IR spectra at different molar compositions of (A) LID:C10, (B) LID:C11, (C) LID:C12, and (D) LID:C13. Red arrows indicate FTIR signals of interest. A black box indicates the carboxylate ion region.

ATR-FTIR was employed to elucidate the nature of molecular interactions within the systems that contributed to the deviation of the experimental data from the solid solubility curves predicted by the Schroeder van Laar model. The stacks of ATR-FTIR spectra of the raw materials and the binary mixtures are shown in Figure 8. The two spectral regions presented display peaks corresponding to the stretching vibrations of the hydrogen bond donor and acceptor groups of the parent materials. A single broad peak observed in the region of 3300–3200 cm^–1^ corresponds to the amide N–H stretching vibration in LID. Upon dilution in the dicarboxylic acids, the peak gradually diminishes. A series of peaks found in the 1800–1500 cm^–1^ region is assigned to carbonyl stretching vibrations. However, carbonyl peak assignment in samples involving dicarboxylic acids is complex due to the presence of free and hydrogen-bonded carboxyl groups, in addition to the presence of multiple polymorphs of odd dicarboxylic acids. Gaussian peak fitting was used to deconvolute the peak overlap observed in the carbonyl region to facilitate a more accurate assignment of peak maxima (see Figure S2).

Deconvolution of the carbonyl signals yielded three bands in all dicarboxylic acids. The major band is found at around 1689 cm^–1^ and is assigned to a hydrogen-bonded C=O stretching vibration. A small shoulder at 1713 cm^–1^ is assigned to a non-hydrogen-bonded carbonyl group. This band is most prominent in odd dicarboxylic acids, possibly due to the coexistence of two different polymorphs within the samples. An additional signal is observed in the region of 1677–1661 cm^–1^ in each of the acids. LID possesses a major peak at 1662 cm^–1^ and a shoulder at 1637 cm^–1^.

The evidence for the formation of intermolecular interactions between LID and C10 comes from the appearance of a peak at 1540 cm^–1^ ascribed to a carboxylate ion asymmetric stretching vibration (Figure 8A). This band is most clearly observed at the 1:1 composition, which indicates the strongest intermolecular attraction. The appearance of this band does not necessarily mean complete acid ionization, and it has been shown previously that carboxylate peaks may also be observed upon partial proton transfer in “poor” ionic liquids and systems with a “confused” proton.^68,69^ An increased prominence of the non-hydrogen-bonded carbonyl band of C10 also supports the formation of intermolecular interactions between LID and C10. The deconvolution of the LID:C11 spectrum reveals processes very similar to the ones observed in the LID:C10 system. However, the carboxylate ion band at 1540 cm^–1^ in Figure 8B is much more prominent in the 1:1 LID:C11 binary mixture, which indicates a greater extent of proton transfer. The semiliquid nature of the ground sample and the appearance of this band suggest a possibility of ionic liquid formation.

In contrast, very weak signals corresponding to a carboxylate stretching vibration are observed in the LID:C12 and LID:C13 systems, thereby indicating that the acids may not interact with LID at the carboxyl sites primarily via a proton transfer. In addition, Figure 8C reveals that the non-hydrogen-bonded signal of the C12 carbonyl group does not increase in prominence upon mixing with LID. This finding suggests that the melting point depression observed in the LID:C12 system and the deviation from the ideal behavior predicted by the Schroeder van Laar model is mainly caused by dipole–dipole interactions and/or van der Waals forces. The interactions in the LID:C13 system are analogous to the interactions observed in the LID:C12 binary mixtures. However, as seen in Figure 8D, the non-hydrogen-bonded carbonyl signal of C13 does become more prominent upon mixing with LID. This is possibly due to the crystal lattice of C13 being broken during mechanical grinding.

## 4 Conclusions

Binary mixtures of LID with C10, C11, C12, and C13 DAs were investigated by thermal, spectroscopic, and crystallographic analysis and theoretical modeling and calculations. It was concluded that LID forms hydrogen-bonding interactions with the DAs investigated, but the interactions are not sufficient for the complete transformation into new homogeneous multicomponent phases. DSC analysis has revealed the formation of eutectic phases in all LID:DA systems. The observed eutectic melting points deviate from the ideal eutectic temperatures calculated by the Schroeder van Laar equation as a result of intermolecular interactions between the reacting components. The appearance of two new endothermic peaks in the DSC thermograms of the LID:C10 system were attributed to a eutectic and an unknown new crystalline phase formation. Mechanochemical grinding induces partial liquefaction of the LID:C11 system, which signifies a possible new phase formation characterized by a lower-than-room-temperature melting point. Intermolecular interactions between LID and the longest DAs, C12 and C13, were found to be the weakest. However, deconvolution of the FTIR spectra has provided evidence for partial ionization in all of the systems investigated. The presence of a single glass transition event in all systems at equimolar LID:DA compositions implies a formation of a homogeneous phase in a supercooled state. The even–odd alternating pattern in physicochemical and crystalline properties that are characteristic of the DA homologous series was observed in the thermal properties of the LID:DA systems. It was demonstrated that eutectic melting points and glass transition temperatures alternate as a function of acid carbon chain length. In addition, a previously undetermined form I of the C13 DA has been solved by SXRD. This work has enhanced our understanding of the influence of increasing dicarboxylic acid chain length on the formation of intermolecular interactions with LID as a strategy to fine-tune physicochemical properties of multicomponent systems for pharmaceutical applications.

## Supporting Information Available

Crystal details and structure refinement for C13 form I; thermograms of LID:C11 samples; and FTIR peak deconvolution (PDF)
