# Supplementary material for: Impact of the Dicarboxylic Acid Chain Length on Intermolecular Interactions with Lidocaine
Source: Mol Pharm. 2022 Jul 19;19(8):2980–91. doi: 10.1021/acs.molpharmaceut.2c00381 (PMC9346613; doi:10.1021/acs.molpharmaceut.2c00381)
Supplement: Supplementary file 1 — mp2c00381_si_001.pdf [file mp2c00381_si_001.pdf]

SUPPORTING INFORMATION

Impact of the dicarboxylic acid chain length on intermolecular interactions with lidocaine

Julija Zotova[1]; Brendan Twamley[2]; Lidia Tajber[1*]

[1] School of Pharmacy and Pharmaceutical Sciences, Trinity College Dublin, College Green, Dublin 2, Ireland

[2] School of Chemistry, Trinity College Dublin, College Green, Dublin 2, Ireland

Table S1. Crystal details and structure refinement for **C13 Form I.**

CCDC No.	2168827
Empirical formula	$C_{13}H_{24}O_4$
M (g/mol)	244.32
T (K)	99.99
Crystal System	monoclinic
SG	C2/c
a (Å)	29.942(18)
b (Å)	4.691(2)
c (Å)	9.597(6)
α (°)	90
β (°)	99.839(18)
γ (°)	90
V (Å^3)	1328.1(13)
Z	4
Dcalc (g/cm^3)	1.222
μ (mm^{-1})	0.723
F(000)	536.0
Crystal size (mm^3)	0.20 × 0.15 × 0.05
Radiation	Cu Kα (λ = 1.54178)
Reflections collected	5630
Independent reflections	1241 R_{int} = 0.0376, R_{sigma} = 0.0338
Data/restraints/parameters	1241/0/82
Goodness-of-fit on F^2 (*S*)	1.141
Final R indexes [$I \geq 2\sigma (I)$]*	R_1 = 0.0397, wR_2 = 0.1194
Final R indexes [all data]	R_1 = 0.0422, wR_2 = 0.1224
Largest diff. peak/hole / e Å^{-3}	0.18/-0.22

*$R_1 = \Sigma ||F_o| - |F_c|| / \Sigma |F_o|$, $wR_2 = [\Sigma w(F_o^2 - F_c^2)^2 / \Sigma w(F_o^2)^2]^{1/2}$.

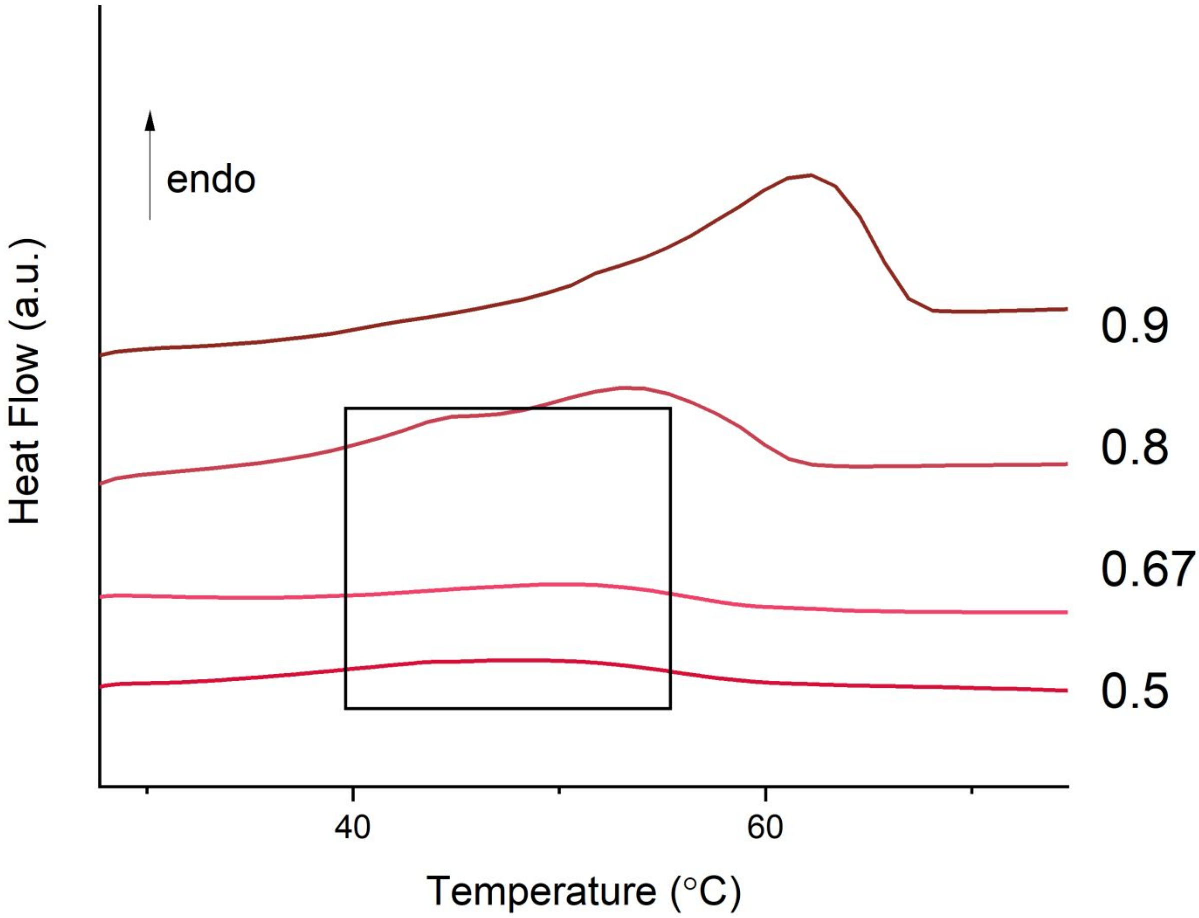

Figure S1. A stack of DSC thermograms for the LID:C11 samples at the range of χ_{LID} = 0.9 – 0.5 molar fractions with the 40 – 60 °C temperature region magnified to show broad endothermic peaks attributed to eutectic meting transitions.

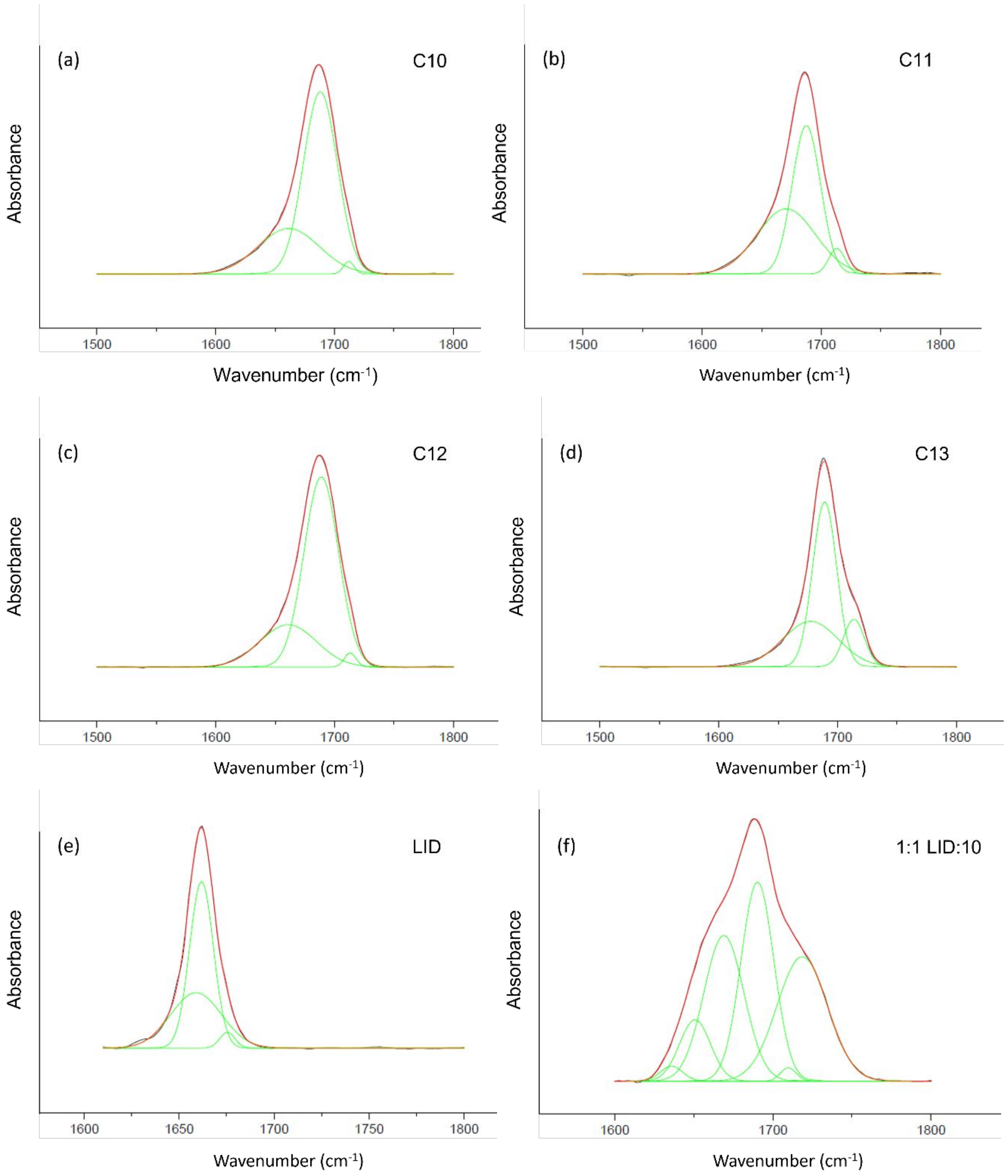
(a)
C10
Absorbance
1500
1600
1700
1800
Wavenumber (cm-1)
(b)
C11
Absorbance
1500
1600
1700
1800
Wavenumber (cm-1)
(c)
C12
Absorbance
1500
1600
1700
1800
Wavenumber (cm-1)
(d)
C13
Absorbance
1500
1600
1700
1800
Wavenumber (cm-1)
(e)
LID
Absorbance
1600
1650
1700
1750
1800
Wavenumber (cm-1)
(f)
1:1 LID:10
Absorbance
1600
1700
1800
Wavenumber (cm-1)

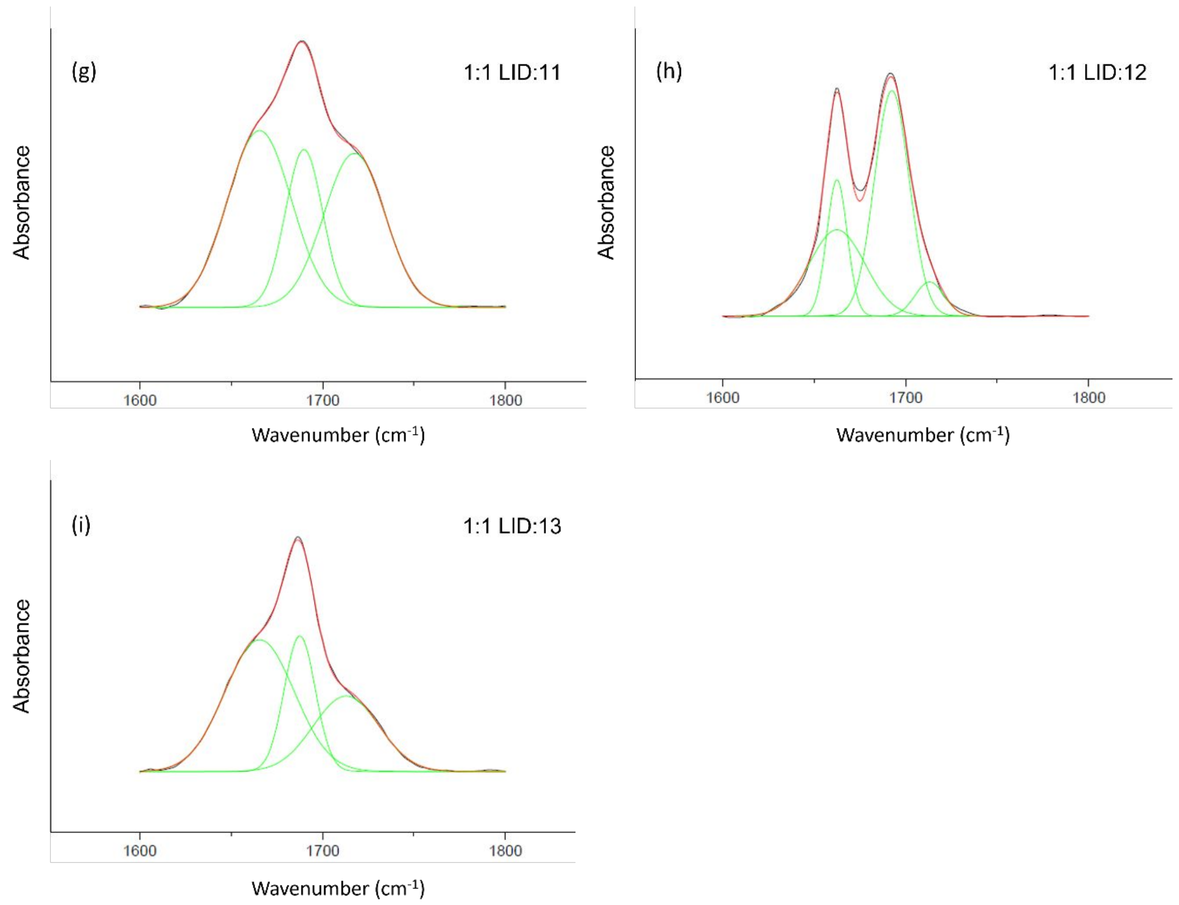

Figure S2. FTIR peak deconvolution of (a) C10, (b) C11, (c) C12, (d) C13, (e) LID, (f) 1:1 LID:C10, (g) 1:1 LID:C11, (h) 1:1 LID:C12 and (i) 1:1 LID:C13. Black line: recorded spectrum; green lines: deconvoluted individual Gauss peaks; and red line: sum of the component peaks.
